# Physiology-guided personalized mechanical ventilation to prevent ventilator-induced lung injury

**DOI:** 10.3389/fmed.2026.1764151

**Published:** 2026-02-12

**Authors:** Raffaele Merola, Denise Battaglini, Marcus J. Schultz, Patricia R. M. Rocco

**Affiliations:** 1 Anesthesia and Intensive Care Medicine, Department of Critical Care, AORN Ospedali Dei Colli, Naples, Italy; 2 Department of Surgical Sciences and Integrated Diagnostics (DISC), University of Genoa, Genoa, Italy; 3 Anesthesia and Intensive Care, IRCCS Ospedale Policlinico San Martino, Genoa, Italy; 4 Department of Intensive Care, Amsterdam University Medical Centres, Amsterdam, Netherlands; 5 Department of Anaesthesia, General Intensive Care and Pain Management, Medical University Wien, Vienna, Austria; 6 Nuffield Department of Medicine, University of Oxford, Oxford, United Kingdom; 7 Mahidol-Oxford Tropical Medicine Research Unit (MORU), Mahidol University, Bangkok, Thailand; 8 Laboratory of Pulmonary Investigation, Carlos Chagas Filho Institute of Biophysics, Federal University of Rio de Janeiro, Rio de Janeiro, Brazil

**Keywords:** acute respiratory distress syndrome (ARDS), mechanical ventilation, respiratory mechanics, respiratory physiology, ventilator-induced lung injury (VILI)

## Abstract

Mechanical ventilation is essential for managing acute respiratory failure, yet it carries a significant risk of ventilator-induced lung injury (VILI). Lung-protective ventilation, most notably through the use of low tidal volumes, has improved outcomes in acute respiratory distress syndrome (ARDS), but these conventional strategies do not fully account for the profound heterogeneity of the injured lung or the variability in patient-specific physiology. Although tidal volumes of 4–8 ml/kg predicted body weight (PBW) provide a general reference for limiting strain, truly protective ventilation requires individualization based on regional aeration, compliance, and recruitability. Variability in these parameters leads to uneven distributions of stress and strain, while dynamic changes in respiratory drive, inspiratory effort, and cardiopulmonary interactions further complicate uniform ventilatory management. The mechanisms underlying VILI: barotrauma, volutrauma, atelectrauma, and biotrauma extend beyond the lung parenchyma and contribute to ventilator-associated diaphragm dysfunction and secondary organ injury. Bedside physiological tools, including esophageal manometry, electrical impedance tomography, and lung ultrasound, allow real-time evaluation of lung stress, regional ventilation, recruitability, and patient effort. When incorporated into clinical decision-making, these modalities facilitate individualized adjustments aimed at avoiding overdistension and collapse, limiting injurious pressures and volumes, and maintaining adequate gas exchange and hemodynamic stability. Advances in technology, such as closed-loop ventilation systems, adaptive control algorithms, and computational modeling, offer additional opportunities to refine personalized strategies and anticipate harmful mechanical patterns. Collectively, physiology-guided, personalized mechanical ventilation shifts practice from protocol-driven approaches to patient-centered care, with the overarching goal of mitigating VILI and improving outcomes in critically ill patients.

## Introduction

Mechanical ventilation (MV) is a cornerstone in the management of acute respiratory failure, including acute respiratory distress syndrome (ARDS) (1). While MV ensures adequate gas exchange, it may also aggravate or precipitate ventilator-induced lung injury (VILI) through excessive mechanical stress and strain (1, 2). The introduction of low tidal volume (VT) ventilation significantly reduced mortality in ARDS (3, 4), yet conventional protective strategies largely rely on global mechanical parameters that do not reflect the profound regional heterogeneity characteristic of ARDS (3).

In ARDS, loss of aerated lung volume, consolidation, gravitational gradients in pleural pressure, and non-uniform alveolar collapse generate steep regional disparities in stress and strain (5). As a result, ventilator settings that appear safe on a whole-lung basis may still impose injurious forces on vulnerable lung regions (6). These structural and functional asymmetries form the central limitation of uniform, population-based ventilation protocols (7, 8).

The increasing availability of bedside physiological monitoring: esophageal manometry, advanced mechanics, gas-exchange profiling, lung ultrasound (LUS), and electrical impedance tomography (EIT) (9, 10) reveals substantial inter- and intra-patient variability in respiratory system behavior (11–13). These tools highlight that respiratory mechanics evolve dynamically with changes in disease severity, hemodynamics, sedation level, and systemic inflammation. Consequently, a physiology-guided, individualized approach to ventilation has gained traction as a strategy capable of addressing regional mechanical stresses more precisely (9).

It is now clear that VILI extends beyond the lungs. Mechanical stress and tissue deformation activate inflammatory pathways leading to systemic dissemination of cytokines, endothelial injury, and multiorgan dysfunction (1, 2, 14). This expanded understanding reinforces the need to refine ventilatory strategies that mitigate both local and systemic consequences of mechanical injury.

Despite substantial advances, fundamental questions remain: which physiological targets best predict benefit, how these measurements should guide bedside decision-making, and whether personalized ventilation improves clinical outcomes. This narrative review synthesizes contemporary physiological principles and emerging evidence supporting individualized mechanical ventilation in ARDS, with emphasis on regional mechanics, mechanical power, patient effort, and the systemic effects of VILI.

## Ventilator-induced lung injury

### Overview of VILI mechanisms

VILI encompasses several interrelated mechanisms: barotrauma, volutrauma, atelectrauma, and biotrauma, which together reflect the lung's response to excessive or unevenly distributed mechanical loads. More recent concepts, such as ergotrauma, viscoelastic stress, and regional mechanical heterogeneity, extend beyond traditional global models and better capture the complex physiology of injured lungs (1, 2, 14).

### Barotrauma and volutrauma: evolving concepts

Early animal studies shaped the foundational understanding of VILI. Webb and Tierney showed that extreme airway pressures caused severe lung injury in rats, but that applying PEEP prevented edema despite identical peak pressures, suggesting that pressure alone was insufficient to cause injury (15). Subsequently, Dreyfuss and colleagues demonstrated that excessive VT was the critical determinant of permeability injury, establishing excessive strain, rather than airway pressure, as the primary driver of volumetric trauma (16).

The “baby lung” model provided a conceptual breakthrough: ARDS reduces the size of the aerated lung available for ventilation (17). A fixed VT distributed over a smaller aerated lung volume results in disproportionately higher regional strain and inflammation. Subsequent computed tomography (CT) studies confirmed that reductions in respiratory system compliance (Crs) in ARDS primarily reflect loss of aerated volume, not increased intrinsic stiffness (18, 19).

Although historically treated as distinct entities, barotrauma and volutrauma are now understood as overlapping manifestations of excessive regional transpulmonary pressure. High airway pressures are not inherently harmful; the relevant distending force is the transpulmonary pressure, which depends on chest wall mechanics and pleural pressure (20, 21).

### Atelectrauma and regional mechanical heterogeneity

Surfactant dysfunction, increased lung weight, and the supine position predispose ARDS patients to regional collapse (22). Cyclic opening and closing of unstable units produce shear forces that disrupt epithelial integrity and amplify inflammation (23). Stress may become several-fold greater at the interface between aerated and collapsed regions, where interdependence magnifies deformation (24).

Microscale heterogeneity is a defining feature of ARDS. Collapsed or partially aerated lung units that undergo intermittent reopening act as local stress multipliers, overdistending adjacent aerated regions through mechanical interdependence and amplification of regional strain (25–27). This mechanism is closely related to the concept of stress concentrators in injured and heterogeneous lungs, whereby abrupt spatial transitions in aeration generate focal increases in stress and strain at the interfaces between collapsed and aerated tissue compartments (28). Such micro-atelectatic regions may remain undetectable on routine imaging yet contribute disproportionately to ventilator-induced lung injury.

PEEP modulates alveolar stability. Adequate PEEP prevents cyclic recruitment, whereas insufficient PEEP increases shear forces and promotes injury (29, 30). Clinically, low VT minimizes the likelihood of exceeding local opening pressures, and appropriately set PEEP stabilizes alveoli throughout the respiratory cycle (31–34). However, excessive PEEP in non-recruitable or focal ARDS may preferentially ventilate already open regions, increasing overdistension and hemodynamic compromise (35, 36).

### Biotrauma and systemic propagation of inflammation

Mechanical deformation triggers mechanotransduction cascades involving cytokine release, reactive oxygen species generation, and endothelial–epithelial disruption (37–39). These processes, collectively termed biotrauma, promote epithelial and endothelial injury, increased permeability, and extracellular matrix remodeling (37, 40–43). Lung-protective ventilation attenuates systemic inflammation and downstream organ dysfunction, underscoring the systemic implications of VILI (37, 40, 43, 44).

### Ergotrauma: the central role of mechanical power

Mechanical power (MP), the energy delivered to the respiratory system per minute, integrates VT, respiratory rate (RR), pressures, and flow, offering a comprehensive measure of mechanical load (45). Although simplified bedside formulas exist, they vary in accuracy and their clinical applicability remains under evaluation (46–50).

Thresholds such as 17 J/min have been proposed but remain debated (51). Observational cohorts associate higher MP with mortality, with approximate thresholds of >17 J/min in mixed ICUs, >22 J/min in ARDS, and >14 J/min in the first 72 h of ECMO support (52–54). Experimental data indicate that MP above 25 J/min can cause structural injury (55).

Driving pressure (ΔP), defined as VT/Crs, remains a robust predictor of mortality and reflects functional lung size (6). The surrogate formula (4 × ΔP) + RR has also been shown to predict outcomes after cardiac arrest (56, 57).

### Viscoelasticity, strain dynamics, and time-dependent injury

The viscoelastic lung exhibits time-dependent stress–strain behavior. For a given VT, faster inflation increases strain rate and may be injurious. Strain is defined as VT normalized to end-expiratory lung volume (EELV); strain rate reflects the velocity of deformation (58). Regional time constants differ widely in ARDS. Units with short time constants fill rapidly, whereas slow units require prolonged inflation. High RR shorten inflation time, increasing heterogeneity and impairing gas exchange. RR has emerged as an independent contributor to VILI and mortality (56). In addition to inspiratory dynamics, emerging experimental evidence highlights the role of expiratory duration in modulating derecruitment. Specifically, shortening expiratory time has been shown to limit cyclic alveolar collapse, preserve end-expiratory lung volume, and reduce regional stress amplification (59). In this context, time-controlled adaptive ventilation (TCAV), a form of airway pressure release ventilation (APRV) that dynamically constrains expiratory duration, has been associated in experimental and computational models with reduced derecruitment and attenuated lung injury (60, 61). Together, these findings support the concept that respiratory rate influences ventilator-induced lung injury not only through its effects on strain rate but also through time-dependent mechanisms governing recruitment–derecruitment dynamics.

The extracellular matrix accommodates small incremental strains, but abrupt increases in VT exceed its capacity to dissipate energy, increasing structural injury risk. Prolonged elevated strain leads to cumulative tissue damage (62).

## Systemic effects of VILI

### Diaphragm dysfunction

**Figure 1:**
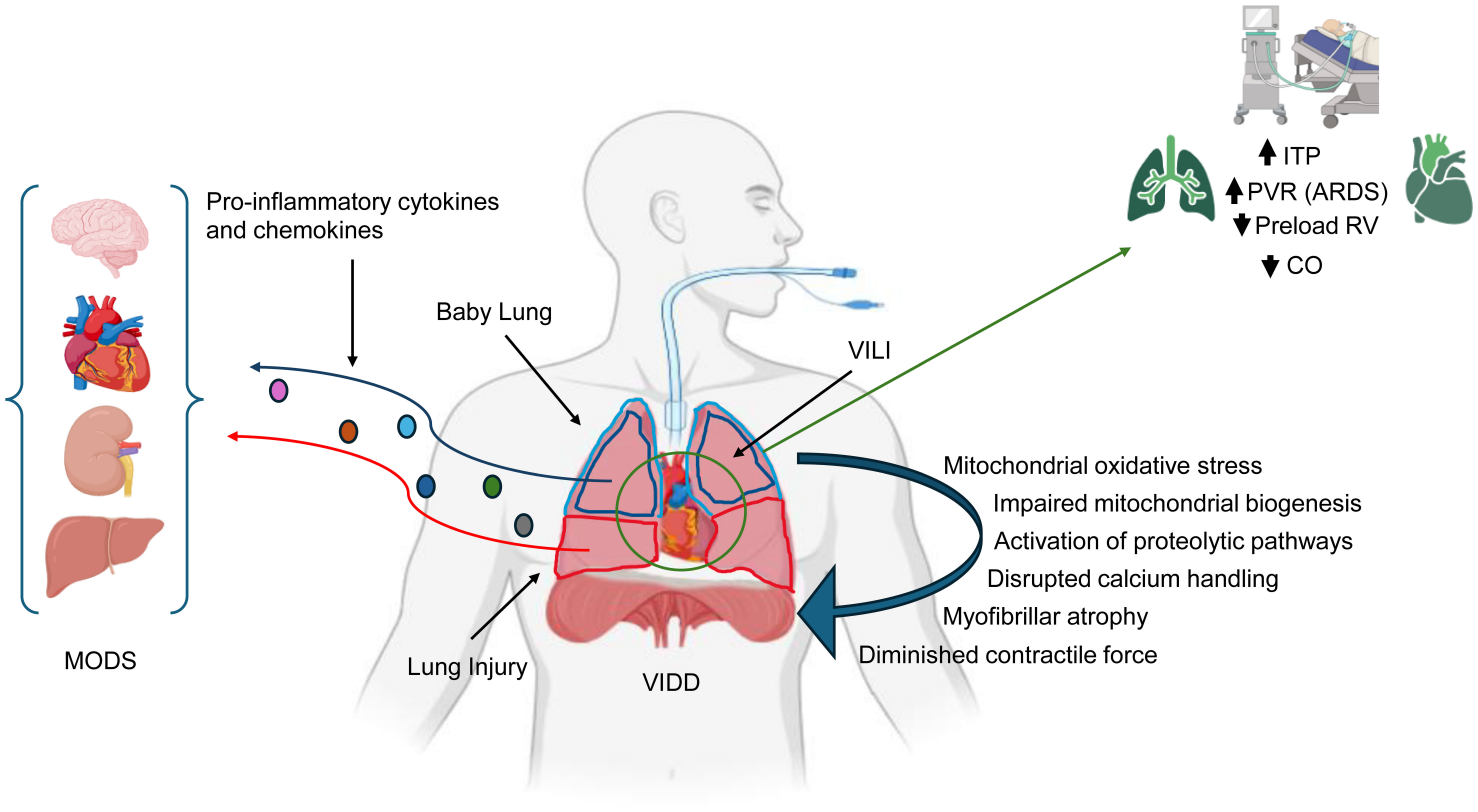
Heart–lung interactions during positive pressure ventilation. Positive pressure ventilation increases intrathoracic pressure (ITP), which can reduce venous return and impair right ventricular (RV) filling, thereby lowering cardiac output (CO) and potentially causing hypotension. These hemodynamic effects are accentuated in severe acute respiratory distress syndrome (ARDS), where pulmonary vascular resistance (PVR) is elevated and RV dysfunction is common. Beyond mechanical effects, ventilator-induced stress can trigger biotrauma, promoting the release of pro-inflammatory cytokines and chemokines into the systemic circulation. This loss of compartmentalization enables inflammatory mediators to reach distant organs, including the brain, cardiovascular system, kidneys, and liver, leading to cellular dysfunction and contributing to multiorgan injury and multiple organ dysfunction syndrome (MODS).

Controlled mechanical ventilation suppresses spontaneous effort and promotes ventilator-induced diaphragm dysfunction (VIDD). Oxidative stress, impaired mitochondrial biogenesis, proteolysis, and disrupted calcium handling contribute to atrophy and weakness, complicating liberation from MV (63, 64) (Figure 1).

### Cardiopulmonary Interactions

Mechanical ventilation substantially alters heart–lung interactions (65). Increases in intrathoracic pressure (ITP) during positive-pressure ventilation decrease venous return, modify right ventricular (RV) preload, and may reduce cardiac output (65–68). These effects are particularly relevant in ARDS, where elevated pulmonary vascular resistance and varying degrees of RV dysfunction are common (69, 70) (Figure 1).

PEEP modulates these relationships in complex ways. Adequate PEEP can lower RV afterload by recruiting collapsed regions and reducing hypoxic vasoconstriction, whereas excessive PEEP increases ITP, limits venous return, and promotes overdistension, worsening RV performance. Fluid status further shapes these interactions: both hypovolemia and fluid overload can magnify adverse hemodynamic effects (71–73).

### Systemic inflammation and organ dysfunction

Mechanical stress at the alveolar–capillary interface activates inflammatory pathways that propagate beyond the lung. Cytokines, chemokines, and damage-associated molecules {e.g. [interleukin (IL)-6, tumor necrosis factor (TNF)-α, IL-1β, IL-8, C-X-C motif chemokine ligand [CXCL]10, CXCL12]} enter the circulation, promoting endothelial activation, glycocalyx disruption, and increased vascular permeability (74). These disturbances impair microcirculatory flow and lead to tissue hypoperfusion. The resulting inflammatory profile resembles early sepsis, even without infection, and represents a key mechanism linking VILI to multiorgan dysfunction (75–77).

### Renal, hepatic, and cardiovascular consequences

Circulating inflammatory mediators impair renal autoregulation, reduce cortical perfusion, and promote tubular injury, increasing the risk of acute kidney injury (78). The liver is similarly affected by inflammation and reduced blood flow, with potential consequences for metabolic and detoxification pathways (79). In the cardiovascular system, inflammatory signaling can depress myocardial contractility, raise pulmonary vascular resistance, and exacerbate RV strain. When combined with inadequate fluid management, these effects may result in pulmonary congestion and systemic venous hypertension (65) (Figure 1).

### Neurological sequelae

Systemic inflammation also affects the brain. Disruption of the blood–brain barrier, alterations in cerebral blood flow related to PaCO_2_ fluctuations, and exposure to sedatives contribute to neuroinflammation and delirium (80). These disturbances may persist long after acute illness, adversely influencing long-term cognitive outcomes (81) (Figure 1).

### Clinical implications

The extrapulmonary effects of VILI highlight the importance of physiology-based ventilation strategies. Approaches that reduce regional alveolar stress, such as low VT ventilation, careful PEEP titration, ΔP limitation, and preservation of spontaneous breathing when appropriate, may mitigate both pulmonary injury and its systemic consequences. Deeper understanding of how mechanical forces propagate through lung tissue and trigger multiorgan dysfunction will be crucial for developing targeted interventions.

## The physiological foundations of personalized ventilation

Modern mechanical ventilation increasingly relies on individualized, physiology-based strategies. The heterogeneity of ARDS, characterized by reduced aerated lung volume, uneven regional compliance, and patchy consolidation, means that global ventilator settings often translate into markedly unequal regional forces. Even within conventionally “protective” ranges, pressures and volumes may impose excessive strain on vulnerable units (1, 5, 7, 8).

Personalized ventilation is grounded in several principles: minimizing injurious mechanical loads, preventing both collapse and overdistension, preserving diaphragm function, and stabilizing cardiopulmonary interactions. These principles stem from an understanding that the “functional lung” in ARDS is considerably smaller than its anatomical volume and behaves as a highly heterogeneous, dynamically evolving structure (17). Achieving lung protection therefore requires tailoring ventilatory support to the individual patient's mechanical properties, recruitability, spontaneous effort, and hemodynamic status (1, 5, 6, 65, 82).

This level of precision requires continuous physiological assessment. Bedside monitoring, via pressure–volume analysis, esophageal manometry, EIT, and LUS, helps characterize regional recruitment, overdistension, *pendelluft*, and ventilation–perfusion matching. These tools are especially valuable during transitions from controlled to assisted ventilation, when the risk of patient self-inflicted lung injury (P-SILI) is highest.

### Respiratory system mechanics and dynamic indexes

Comprehensive evaluation of respiratory mechanics is essential for defining protective ventilator targets. Crs reflects the size of the aerated “baby lung” in ARDS more than total lung volume. Low Crs indicates a smaller ventilated lung and increased susceptibility to overdistension, even when VT is within the 4–8 ml/kg PBW range (5).

While VT normalized to PBW is reasonable for patients with structurally normal lungs, the height–lung size relationship underpinning PBW does not apply in ARDS. No reliable bedside technique exists to precisely quantify EELV, and its clinical utility remains uncertain. Crs may therefore provide a more physiologically meaningful estimate of functional lung size. Conceptually, normalizing VT to Crs is equivalent to normalizing it to ΔP. Airway resistance (Raw) influences flow distribution and alveolar pressure dynamics. Elevations in Raw worsen heterogeneity, increase intrinsic PEEP, and promote dynamic hyperinflation, especially at high RR (9, 83). Driving pressure has emerged as a robust marker of lung stress (84). Observational studies show that ΔP < 15 cmH_2_O, and ideally < 10 cmH_2_O, is associated with improved survival (85). The STAMINA trial tested a ΔP-limited strategy incorporating Crs-optimized PEEP and VT titration, improving oxygenation, mechanics, and VILI biomarkers but not ventilator-free days (86). The trial's early termination and brief intervention period limit interpretation, but its findings reinforce the central importance of early ΔP management (86). MP provides an integrative measure of mechanical load, but its clinical role continues to evolve (45).

Dynamic indices refine assessment of patient–ventilator interactions. Esophageal pressure monitoring estimates transpulmonary pressure, separating chest-wall from lung stress, especially relevant in patients with altered chest-wall mechanics. Measures of respiratory effort, including the esophageal pressure swing (ΔPes), airway occlusion pressure (P0.1), and diaphragmatic thickening fraction on ultrasound, help identify excessive spontaneous effort that could precipitate P-SILI (1, 82). Synchrony assessment identifies double triggering, breath stacking, and ineffective efforts, all linked to increased work of breathing, lung stress, and diaphragm injury (82, 87).

Recruitability assessment is central to individualized PEEP titration. Tools including pressure–volume curves, EIT-derived regional compliance, LUS re-aeration scoring, and gas-exchange changes during PEEP trials help determine whether additional PEEP will reopen collapsed tissue or induce overdistension (88, 89).

## Heterogeneity in lung structure and function

Lung heterogeneity is a defining characteristic of ARDS and the primary rationale for individualized ventilation. Recruitability varies widely between patients. Those with highly recruitable, typically non-focal patterns may benefit from recruitment maneuvers and higher PEEP to reopen collapsed units and promote homogeneous ventilation. Conversely, individuals with focal or minimally recruitable disease are prone to overdistension and hemodynamic compromise when exposed to aggressive recruitment or high PEEP (90, 91).

The LIVE study illustrated this concept: misclassification of lung morphology resulted in inappropriate recruitment strategies and worse outcomes (92). Thus, bedside assessment of recruitability is essential. Tools such as the recruitment-to-inflation ratio, esophageal manometry, LUS, CT, and EIT offer complementary insights to balance recruitment against overdistension. The overarching goal is to achieve the most homogeneous ventilation pattern possible while minimizing regional stress and strain (73).

## Respiratory drive and effort

Respiratory drive and inspiratory effort critically influence lung stress, synchrony, and diaphragm function during assisted ventilation. Excessive effort generates large negative pleural swings, increasing transpulmonary pressure and predisposing to P-SILI (13). Insufficient effort, in contrast, accelerates diaphragm disuse atrophy and prolongs weaning (93).

Monitoring neural, mechanical, and ventilatory parameters helps define an optimal “effort safe zone.” Diaphragm electrical activity (EAdi) provides continuous measurement of neural drive; although absolute values vary among patients, trends over time are clinically informative (94–98). Airway occlusion pressure (P0.1) reflects global neural drive and is independent of muscle fatigue or airway resistance (99, 100). Elevated P0.1 correlates with higher effort, dyspnea, prolonged ventilation, and increased mortality (101, 102).

Esophageal manometry allows direct quantification of pleural pressure swings, enabling estimation of inspiratory muscle pressure (Pmus) and identification of excessive or insufficient effort (45). The airway pressure deflection during an end-expiratory occlusion (ΔPocc) provides a practical surrogate for Pmus, with extreme values associated with worse outcomes (103). Diaphragm ultrasound adds further insight; a thickening fraction of approximately 30% often marks excessive effort, though considerable inter-individual variability persists (104).

Optimizing the alignment between patient effort and mechanical assistance is central to preserving diaphragm function and preventing occult lung stress. Achieving this balance requires integrating respiratory mechanics, recruitability, chest-wall compliance, and hemodynamics.

## Physiology-guided protective strategies

Physiology-guided ventilation represents a shift from fixed, population-based targets toward individualized settings informed by mechanical, regional, and biological cues. Its goal is to define, for each patient, a protective window that minimizes VILI, diaphragm injury, and hemodynamic compromise.

### PEEP and recruitment

PEEP stabilizes alveoli and reduces cyclic collapse, yet excessive levels can cause regional overdistension and reduce cardiac output. Trials comparing higher vs. lower PEEP strategies: ART (105), ALVEOLI (106), ExPress (107), and LOV (33), have produced inconsistent results, reflecting heterogeneity in patient characteristics, recruitability, and disease severity. A meta-analysis of ALVEOLI, ExPress, and LOV suggested a potential benefit in severe ARDS (108), although aggressive recruitment maneuvers remain associated with well-recognized risks.

Driving pressure provides a pragmatic tool for PEEP optimization, with the protective range often corresponding to the lowest ΔP (109). However, ΔP must be interpreted cautiously: alterations in lung volume, chest-wall elastance, airway closure, and intratidal recruitment can modify its relationship to true transpulmonary stress (9, 110).

Transpulmonary pressure estimated by esophageal manometry offers a more direct assessment of lung-distending forces and separates lung from chest-wall contributions (111, 112).

## Imaging-based individualization

Imaging tools provide complementary insights for tailoring ventilator settings. CT remains the reference standard for characterizing lung morphology, quantifying recruitability, and identifying focal vs. non-focal patterns, though its use is limited by the need for transport and radiation exposure (19, 35, 113–123). EIT offers continuous bedside assessment of regional ventilation, enabling early detection of overdistension or collapse and supporting titration toward more homogeneous ventilation (124, 125). Evidence from clinical studies is mixed: some report reduced PEEP requirements or survival benefits compared with pressure–volume loop guidance, while broader use is constrained by equipment availability, operator dependency, and the need for consistent belt positioning (126–128). Lung ultrasound provides a practical, radiation-free assessment of aeration, consolidation, and PEEP responsiveness, assists in classifying ARDS phenotypes, and can estimate opening and closing pressures during recruitment maneuvers (129–131).

## Diaphragm-protective ventilation

Preserving diaphragmatic function has become a critical complement to lung protection. Maintaining inspiratory effort within a physiological range prevents both disuse atrophy from excessive unloading and injurious transpulmonary swings from uncontrolled spontaneous effort (63, 64). Continuous assessment of inspiratory effort supports fine-tuning of ventilatory assistance and helps balance respiratory drive with lung protection (132).

Early mobilization and structured reintroduction of spontaneous breathing, particularly with proportional modes, help retain contractility and maintain safe transpulmonary pressures. These strategies are especially important during the transition from controlled to assisted ventilation, when both diaphragm injury and occult lung stress are most likely (133–135).

## Cardiovascular integration

Physiology-guided ventilation must incorporate cardiovascular considerations, recognizing the close interaction between intrathoracic pressures, preload, afterload, and RV function. Positive pressure ventilation alters venous return and pulmonary vascular resistance, effects that are amplified in ARDS due to elevated RV afterload and impaired RV-pulmonary arterial coupling.

Volume status critically modulates these interactions: hypovolemia exacerbates the reduction in venous return induced by positive pressure, whereas fluid overload increases RV preload and pulmonary vascular congestion, intensifying afterload on the failing RV. Thus, fluid stewardship is inseparable from ventilator titration. Adjustments to PEEP or ΔP must be interpreted in the context of hemodynamic reserve and preload responsiveness (8, 65–73).

Echocardiography and dynamic responsiveness indices (PPV, PLR-induced SV change) provide essential information on RV size, septal motion, and pulmonary pressures, helping identify patients at risk of hemodynamic compromise during ventilator titration (136–139).

## Technological and conceptual advances enabling personalization

Recent technological innovations and growing understanding of ARDS pathophysiology have accelerated the shift from standardized protocols to individualized, physiology-driven ventilation. These advances support multimodal evaluation of lung mechanics, regional heterogeneity, inspiratory effort, and cardiopulmonary interactions to guide patient-specific adjustments.

Bedside monitoring tools, adaptive ventilation systems, integrated data platforms, and computational models now provide an unprecedented level of granularity for tailoring support to the evolving characteristics of the injured lung.

The technologies and monitoring approaches discussed in this review were selected to illustrate physiologically grounded and clinically relevant pathways toward personalized mechanical ventilation, rather than to provide an exhaustive inventory of available tools. As the field continues to evolve, additional methodologies and innovations will likely further expand and refine this framework.

## Bedside monitoring and effort assessment

Tools for continuous assessment of Crs, ΔP, and inspiratory effort have matured considerably. Combined with advanced hemodynamic monitoring, they allow clinicians to quantify stress and strain, identify excessive respiratory drive, detect hemodynamic vulnerability, and titrate support within lung-, diaphragm-, and heart-protective domains (140). These features are particularly valuable in ARDS, where respiratory mechanics and cardiopulmonary status can evolve rapidly.

## Closed-loop and adaptive ventilation systems

Closed-loop systems represent an important technological step forward. By integrating variables such as VT, RR, airway pressures, gas exchange, and dynamic respiratory mechanics, these systems adjust ventilator settings automatically in response to patient demand. Incorporation of parameters related to VILI risk enables maintenance of protective targets with fewer manual interventions (141, 142).

A systematic review of 51 randomized trials demonstrated that automated ventilation reduces clinician workload and performs comparably to expert clinicians in adjusting ventilator parameters (142). Automated oxygen-control systems increase time within target SpO_2_ ranges and reduce staff burden relative to manual titration (143). Whether these technologies improve patient-centered outcomes remains uncertain and requires rigorous evaluation.

Importantly, in this section the term “adaptive ventilation” is used in a specific sense to denote algorithm-driven closed-loop systems, rather than clinician-directed ventilation strategies based on protocolized adjustments.

## Computational modeling and machine learning

Emerging computational tools, including physiologic modeling, parameter estimation, and machine learning, offer new opportunities to further refine individualized ventilatory care (144, 145). Recent advances have enabled models of varying complexity to be calibrated directly to patient-specific or experimental data, allowing a more detailed characterization of lung mechanics and time-dependent behavior. In principle, such approaches could support real-time assessment of regional mechanics, energy transfer, and spatial heterogeneity, thereby informing more precise ventilator adjustments and potentially reducing the risk of VILI (146, 147). However, clinical application remains at an early stage, and robust prospective validation is essential before widespread implementation.

## Biological subphenotypes and precision medicine

Advances in ARDS subphenotyping also represent an important step toward precision medicine (148). Latent class analyses across multiple RCTs consistently identify biological subphenotypes, notably hyperinflammatory and hypoinflammatory, characterized by distinct inflammatory, endothelial, and coagulation profiles (149–153).

The hyperinflammatory subphenotype, marked by high cytokine levels, endothelial disruption, more severe shock, and metabolic acidosis, carries substantially higher mortality. Importantly, these subphenotypes respond differently to ventilatory, fluid, and pharmacologic strategies: patients with the hyperinflammatory phenotype appear to benefit from higher PEEP, conservative fluid management, and immunomodulatory therapies, including corticosteroids, whereas those with the hypoinflammatory phenotype derive less benefit and may be harmed by these same interventions (149–153).

These divergent responses parallel functional and radiographic distinctions between recruitable and non-recruitable lung morphologies, underscoring the importance of integrating physiologic assessment with biomarker-based classification. Prospective trials will be essential to determine whether subphenotype-guided strategies translate into improved outcomes.

## Clinical translation and future perspectives

Although understanding of VILI has expanded substantially, key uncertainties persist. Lung-protective ventilation, particularly low VTs volumes and higher PEEP, remains central, yet survival benefits vary, especially regarding PEEP titration (33, 105–108). Newer approaches targeting lower ΔP and reduced MP offer additional protection (51–57, 84–86), but uniform application of low tidal volumes is unlikely to benefit all patients, and PEEP must be individualized to avoid overdistension or hemodynamic instability. These limitations reinforce the need for strategies grounded in each patient's unique physiology.

Despite conceptual progress, several barriers impede clinical translation. Specialized monitoring tools, the need for precise calibration and placement, and cost considerations restrict widespread adoption. Many clinicians remain unfamiliar with interpreting advanced physiologic metrics or integrating multimodal data into moment-to-moment decisions. Continuous monitoring and frequent adjustments must also be coordinated with parallel ICU therapies, a challenge in high-acuity or resource-limited settings. Overcoming these barriers will require targeted training, improved user interfaces, and coordinated decision-support systems.

Personalized ventilation depends on integrated practice across the multidisciplinary ICU team. Intensivists, respiratory therapists, physiotherapists, and nurses must jointly interpret physiologic data, balance patient effort against lung protection, and manage transitions between ventilation modes safely (133, 134). Such collaboration is essential to avoid under- or over-assistance, minimize injury to both lung and diaphragm, and maintain hemodynamic stability.

**Figure 2:**
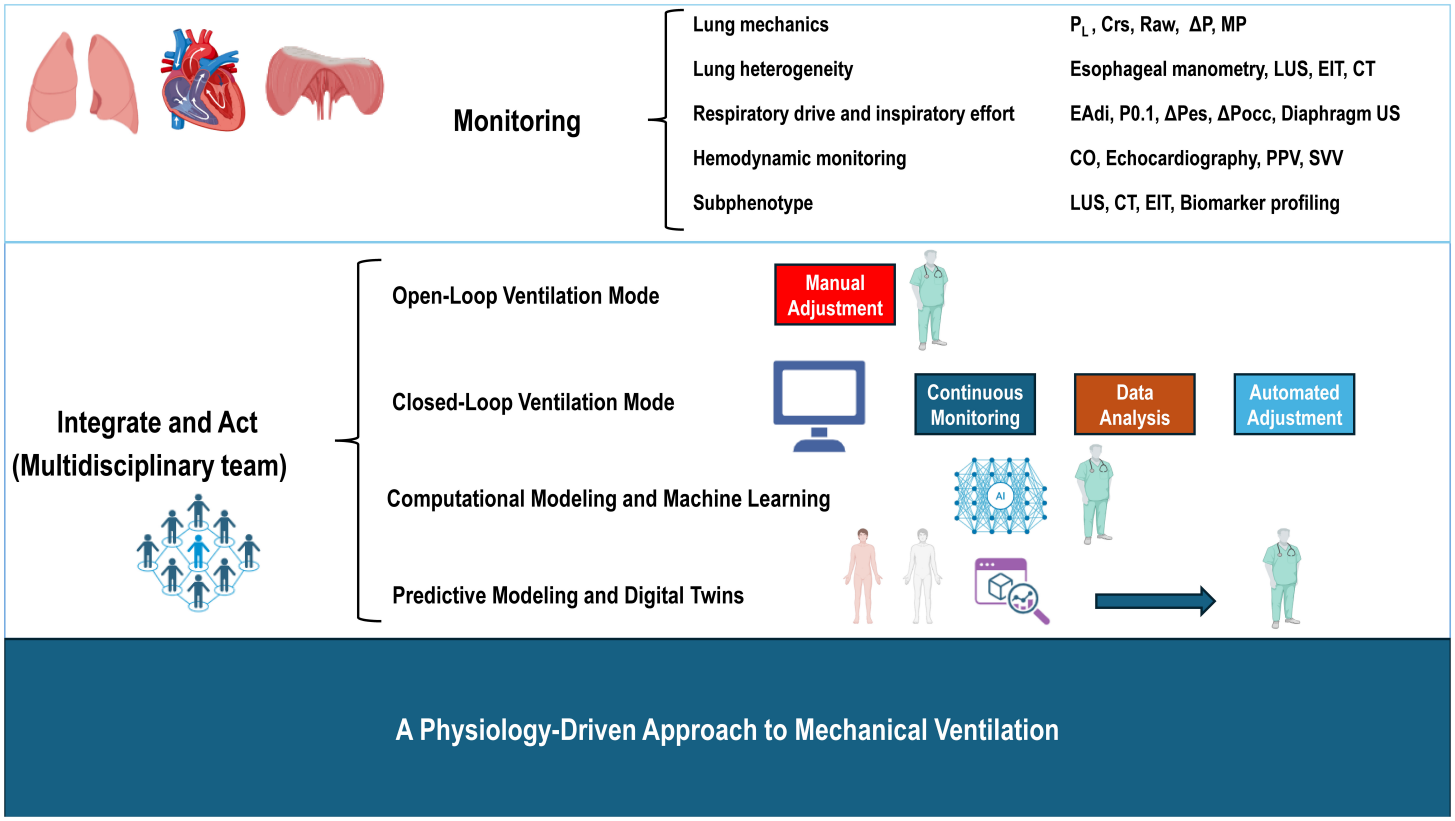
From monitoring to personalization: a physiology-driven approach to mechanical ventilation. Continuous monitoring of lung mechanics, patient respiratory effort, and advanced hemodynamic parameters provide real-time insight into pulmonary stress and strain, enabling early recognition of injurious breathing patterns and supporting ventilatory management within lung-, diaphragm-, and heart-protective ranges. The effective use of physiology-guided, individualized ventilation depends on coordinated interpretation of these data by the multidisciplinary team to balance patient effort with lung protection and to ensure safe transitions between controlled and assisted modes. Emerging technologies further strengthen this personalized approach: adaptive and closed-loop systems adjust ventilator settings in response to patient-specific changes, whereas machine-learning tools and digital-twin models integrate imaging, biomarkers, and physiologic signals to detect harmful trends, simulate treatment responses, and suggest optimal strategies. Together, these innovations shift clinical practice from reactive modification of ventilator settings to proactive, physiology-based optimization tailored to the individual patient. CO, cardiac output; Crs, respiratory system compliance; CT, chest computed tomography scan; EAdi, electrical activity of the diaphragm; EIT, electrical impedance tomography; LUS, lung ultrasound; MP, mechanical power; P0.1, airway occlusion pressure at 0.1 s; PL, transpulmonary pressure; PPV, pulse pressure variation; Raw, airway resistance; SVV, stroke volume variation; ΔP, driving pressure; ΔPocc, airway pressure deflection during an end-expiratory occlusion; ΔPes, esophageal pressure swing; and US, ultrasound.

Predictive modeling and digital-twin technologies, patient-specific simulations of lung mechanics and physiology, represent the next major step (154). By integrating demographic, imaging, biomarker, and real-time physiologic data, these tools may forecast regional stress patterns, guide ventilator selection, and anticipate responses to therapeutic changes, enabling more anticipatory care (154, 155). Figure 2 shows an approach to mechanical ventilation based on physiology.

Ultimately, the goal is to prevent VILI rather than respond to its consequences. Achieving this will require the integration of advanced monitoring, predictive analytics, and adaptive ventilator adjustments to maintain regional stress and strain within safe limits while preserving diaphragmatic function and cardiopulmonary stability. Effective implementation will depend on rigorous trials, seamless incorporation into ICU workflows, and broad training to ensure that physiology-guided ventilation becomes standard practice rather than a specialized approach.

## Conclusion

A comprehensive understanding of respiratory physiology remains fundamental to preventing iatrogenic injury during mechanical ventilation. Recognizing the heterogeneity of lung mechanics, patient effort, and cardiopulmonary interactions allows clinicians to tailor ventilatory support that minimizes lung and diaphragm injury while ensuring adequate gas exchange. Personalized ventilation represents a transition from uniform, protocolized strategies to support that is dynamically adapted to the patient's evolving physiology. As multimodal monitoring, advanced analytics, and computational modeling become increasingly integrated into clinical care, they will strengthen the foundation for proactive, physiology-guided interventions that anticipate injury, preserve organ function, and ultimately improve outcomes in critically ill patients.
